# Screening, diagnosis and management of hyperthyroidism in pregnancy: Number 8 – Agosto 2022

**DOI:** 10.1055/s-0042-1756521

**Published:** 2022-09-08

**Authors:** Carlos Alberto Maganha, Rosiane Mattar, Cleo Otaviano Mesa Júnior, Suemi Marui, Sara Toassa Gomes Solha, Patrícia de Fátima dos Santos Teixeira, Alberto Carlos Moreno Zaconeta, Renato Teixeira Souza

**Affiliations:** 1 Faculdade de Ciências Médicas de São José dos Campos, São José dos Campos, SP, Brazil; 2 Departamento de Obstetrícia, Escola Paulista de Medicina, São Paulo, SP, Brazil; 3 Universidade Federal Paraná, Curitiba, PR, Brazil; 4 Faculdade de Medicina, Universidade de São Paulo, São Paulo, SP, Brazil; 5 Policlínicas Municipal, Sorocaba, SP, Brazil; 6 Universidade Federal do Rio de Janeiro, Rio de Janeiro, RJ, Brazil; 7 Universidade de Brasília, Brasília, DF, Brazil; 8 Universidade Estadual de Campinas, Campinas, SP, Brazil

## Key points

- The physiological changes of pregnancy that interfere with the production, release and availability of the active form of hormones interfere with the diagnosis and management of hyperthyroidism during pregnancy.
- Gestational thyrotoxicosis or transient hyperthyroidism, the most common cause of hyperthyroidism in pregnancy, is related to the increased production of human chorionic gonadotropin (hCG) and may persist until week 18.
- Untreated hyperthyroidism can have fetal, neonatal, and maternal effects.
- The obstetrician must be aware of the fetal, neonatal and/or maternal risks caused by the drug treatment of hyperthyroidism during pregnancy.
- Graves’ disease (GD) is the main pathology etiologically associated with hyperthyroidism in pregnancy.
- The diagnosis of hyperthyroidism in pregnancy is preferably made by measuring free thyroxine (FT4) and thyroid-stimulating hormone (TSH).
- The measurement of anti-TSH receptor antibody (TRAb) allows the diagnosis of GD, which is an important cause of hyperthyroidism.
- Propylthiouracil (PTU) is the first-choice drug for the treatment of hyperthyroidism in pregnancy in the first trimester, while methimazole (MMZ) is used in the second and third trimesters and puerperal period.
- The use of antithyroid drugs (ATD) is allowed during breastfeeding.
- Radioactive iodine ( ^131^ I) should not be used during pregnancy or breastfeeding.

## Recommendations

- The diagnosis of hyperthyroidism should be confirmed when TSH is suppressed (<0.1 mUI/L) and FT4 is above the upper limit of normality of the lab kit used.
- The use of ATDs for the treatment of gestational thyrotoxicosis or subclinical hyperthyroidism is not recommended.
- In case of suspicion of gestational thyrotoxicosis, whenever possible, GD should be ruled out with the measurement of TRAb.
- All overt hyperthyroidism should be treated during pregnancy, given the fetal, neonatal and maternal risks of the disease.
- The doses of ATDs (PTU and MMZ) should be the lowest possible to maintain FT4 levels at the upper limit of normality of the lab kit used, considering the passage of these medications through the placenta and the risk of fetal hypothyroidism.
- The recommended doses of PTU are 200-400 mg daily, divided into three daily intakes. Propylthiouracil has less teratogenicity and greater side effects than MMZ. It is preferably used in the preconception period and in the first trimester of pregnancy. Methimazole, in turn, is recommended in doses of 10-30 mg daily, taken in a single dose. Methimazole is preferably used in the second and third trimesters of pregnancy, and during breastfeeding.
- Pregnant women with hyperthyroidism should be followed up in high-risk antenatal care. Barring other complications, they should have follow-up appointments every other week until week 28 and weekly visits from then until delivery.
- Treatment of the hyperthyroid phase of postpartum thyroiditis with ATDs is not recommended.

## Background

During pregnancy, there is an increase in the stimulation of the hypothalamic-pituitary-thyroid axis through different mechanisms: 1

- Increase in the serum concentration of estrogens, accompanied by an increase in thyroid hormone binding globulin (TBG - thyroxine binding protein) and consequent reduction in the free fractions of thyroid hormones (THs);
- Greater iodine clearance;
- Greater degradation of THs by placental deiodinases;
- Increase in the serum concentration of hCG that stimulates the thyroid tissue by cross-reacting with the TSH receptor, which can generate goiter and transient gestational hyperthyroidism.

**Figure 1.**
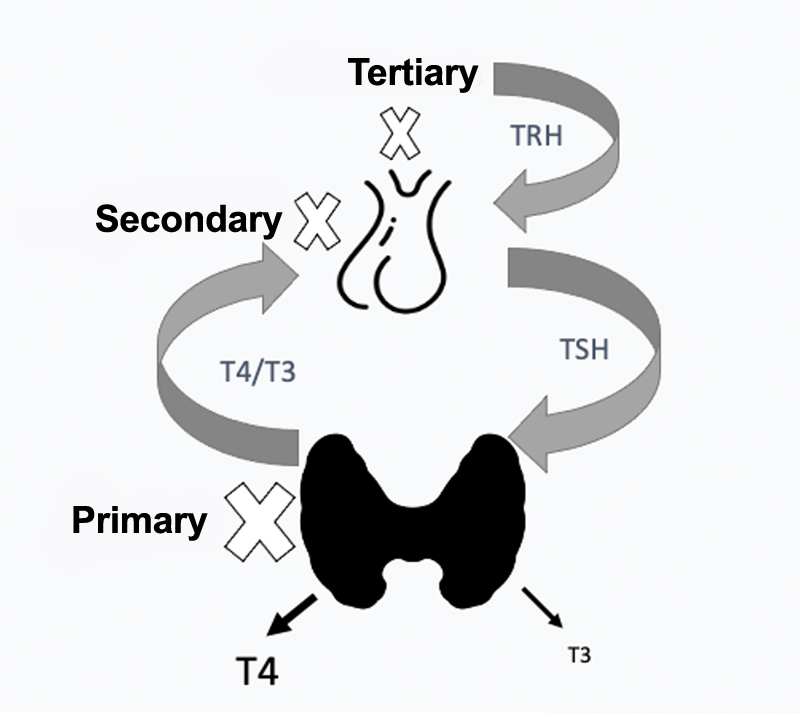
Schematic representation of the hypothalamic-pituitary-thyroid axis indicating the types of hypothyroidism. TRH: thyrotropin releasing hormone; TSH: thyroid stimulating hormone; T4: thyroxine; T3: triiodothyronine. Source: prepared by the Working Group for Thyroid Dysfunctions in Pregnancy (Brazilian Society of Endocrinology and Metabology – SBEM).

This stimulus to the axis explains why pregnant women, especially in the first trimester, have lower TSH concentrations than non-pregnant women. 1 Metabolic demands are greater in the first trimester of pregnancy, a critical period for the occurrence of thyroid dysfunctions, given the changes in the stimulation of the thyroid gland. All changes described in the physiology of the hypothalamic-pituitary-thyroid axis ensure the supply of TH to the fetus, especially in the period when the fetal thyroid is not functionally mature yet. Although the development of the gland begins at week 8, it functions fully only between weeks 18-20, therefore, until that moment, the fetus is totally dependent on the placental transfer of maternal THs. There is a compensatory mechanism via feedback between the thyroid, pituitary and hypothalamus that regulates glandular functioning. Knowing this mechanism helps to find the cause of a possible dysfunction ( Figure 1 ).

Due to the previously mentioned physiological changes during pregnancy, the reference values for TSH, FT4 and total T4 (T4T) must be adjusted. 2 In relation to TSH, this means that if we reduce 0.4 mU/L of the lower reference limit stated by the lab kit, the TSH level for the diagnosis of hyperthyroidism would be <0.1 mU/L for most laboratories. This is a recommendation from the American Thyroid Association (ATA) and proved to be adequate as a determinant of the specific reference range for pregnant women in a recent study of a healthy population in Rio de Janeiro. 2 3 Thyrotoxicosis is a clinical syndrome characterized by hypermetabolism and hyperactivity resulting from exposure to excessive amounts of THs. The main cause of thyrotoxicosis is the hyperfunction of the thyroid gland, called hyperthyroidism, which is characterized in the laboratory by a reduction in TSH levels with or without an increase in TH concentration. 4

## How can hyperthyroidism present during pregnancy and what are its main etiologies?

Grave’s disease of autoimmune etiology is the most common pathological cause of hyperthyroidism in pregnancy, representing 95% of cases. It occurs in 0.4-1% of women before pregnancy and in approximately 0.2% during pregnancy. Gestational transient thyrotoxicosis (GTT) or transient gestational hyperthyroidism, is the main differential diagnosis of GD and the most common clinical condition, occurring in up to 5% of pregnancies and limited to the first half of pregnancy. This condition is characterized by a reduction in TSH, with or without an increase in FT4. Elevated hCG at the beginning of pregnancy is the cause of thyroid stimulation, causing mild and transient hyperthyroidism, which may be associated with hyperemesis gravidarum. There is a greater risk for GTT in conditions of high hCG concentration, such as twin pregnancy, hydatidiform mole, and choriocarcinoma. 4

Other causes of hyperthyroidism in pregnancy are toxic adenoma, subacute thyroiditis, multinodular or iatrogenic goiter due to excessive ingestion of THs. 2 5 Hyperthyroidism can manifest itself in pregnancy as:

- **Overt hyperthyroidism** , characterized by reduced TSH and elevated TH levels, especially FT4;
- **Subclinical hyperthyroidism** , characterized by reduced TSH and normal THs.

**Figure 2.**
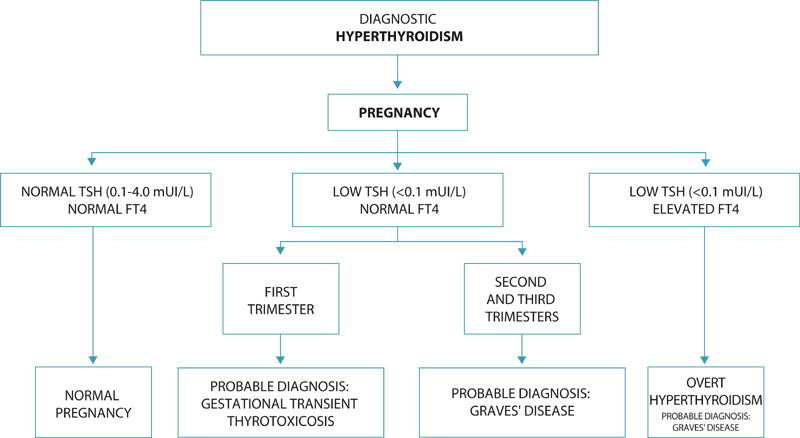
Diagnostic hyperthyroidism. TSH: thyroid stimulating hormone; FT4: free thyroxine. Source: Prepared by the Working Group for Thyroid Dysfunctions in Pregnancy (CNEGAR and Brazilian Society of Endocrinology and Metabology – SBEM).

The possible scenarios in pregnant women with hyperthyroidism are correlated with the time of diagnosis, previous and/or current treatment and the quality of metabolic control ( Chart 1 ) ( Figure 2 ).

**Chart 1:** Diagnoses related to hyperthyroidism in pregnancy that may impact clinical management

	Diagnosis	TSH	T4L	TRAB
Hyperthyroidism diagnosed prior to pregnancy	GD in remission	Normal	Normal	Not necessary
	GD after treatment with RAI or surgery, on FT4 therapy	Normal	Normal	Request
	Controlled GD in use of low doses of ATD	Normal	Normal	Request
	Uncontrolled GD on high doses of ATD	Low	Elevated	Request
	Other causes of hyperthyroidism (TMNG or TNG	Normal or reduced	Normal or elevated	Not necessary
Hyperthyroidism diagnosed in pregnancy	Subclinical hyperthyroidism Probable diagnosis: GTT	<0.1 mU/L	Normal	Request only if there is suspicion of GD
	Clinical hyperthyroidism Probable diagnosis: GD	<0.1 mU/L	Elevated	Request

TSH: thyroid stimulating hormone; FT4: free thyroxine; T4T: total thyroxine; T3T: total triiodothyronine; GD: Graves’ disease; RAI: radioactive iodine therapy; LT4: levothyroxine; ATD: antithyroid drug; TMNG: toxic multinodular goiter; TNG: toxic nodular goiter; GTT: gestational transient thyrotoxicosis

Source: Prepared by the Working Group for Thyroid Dysfunctions in Pregnancy (CNEGAR and Brazilian Society of Endocrinology and Metabology – SBEM).

## Hyperthyroidism with diagnosis prior to pregnancy

- *GD in remission:* when the pregnant woman was diagnosed with GD, underwent clinical treatment with an ATD and is in remission after discontinuing the drug, maintaining normal thyroid function. This patient should have her thyroid function monitored during pregnancy and if normal, there is no need for additional fetal testing or monitoring. The postpartum period requires special care given the higher risk of GD recurrence and postpartum thyroiditis.
- *GD after treatment with*^131^*I or surgery:* when the patient needs replacement with levothyroxine. Monitoring of TRAb is necessary, as it may be elevated even after treatment, which increases the risk of fetal hyperthyroidism via placental passage.
- *GD using low doses of ATD:* when the patient being treated for GD with low doses of ATD – 5-10 mg of MMZ or 50-200 mg of PTU – is under control and wants to become pregnant or is pregnant. Antithyroid drugs may be withheld depending on recurrence risk factors.
- *GD using high doses of ATD:* when the patient has difficulty controlling hyperthyroidism, definitive therapy with ^131^ I or surgical excision may be recommended before pregnancy. If she becomes pregnant inadvertently, the recommendation is to maintain the drug.
- *Patients with hyperthyroidism due to other causes such as toxic multinodular or nodular goiter:* in this situation, definitive treatment is suggested before pregnancy and if the patient becomes pregnant before that, it is recommended to maintain drug therapy without the need for TRAb monitoring.

## Hyperthyroidism diagnosed during pregnancy

- *Subclinical hyperthyroidism* presents with TSH < 0.1 mIU/L and normal FT4, and the diagnosis is laboratory confirmed. The patient should not be treated with ATD and the differential diagnosis between GTT and GD is important.
- *Overt hyperthyroidism* presents with TSH < 0.1 mIU/L and elevated FT4. The most likely diagnosis is GD. It is important to define the etiology with the measurement of TRAb to evaluate treatment and perform fetal monitoring.

## Which symptoms in pregnant women should make the health professional suspect hyperthyroidism?

The signs and symptoms of hyperthyroidism are linked to a clinical state of hypermetabolism. A hypermetabolic state should be suspected when the pregnant woman has palpitations and/or tachycardia, irritability, aggressiveness, restlessness, fine tremors, sweating, hot skin, and weight loss or weight gain less than expected. Evidently, as these are non-specific symptoms, they can go unnoticed, particularly during pregnancy. More specific findings include goiter, ophthalmopathy and pretibial myxedema suggestive of GD. 6 7

## Which TSH and FT4 values make the diagnosis of hyperthyroidism?

The laboratory diagnosis of hyperthyroidism during pregnancy is defined as reduced TSH and elevated FT4 levels using specific reference values for pregnant women. 8 Note that reference values for thyroid function during pregnancy are different because of the typical physiological changes of pregnancy and the characteristics of laboratory assays used in this evaluation. Furthermore, iodine sufficiency in the population and race can also influence these parameters. Specialist societies recommend that reference values for pregnant women are determined for each population according to each trimester and using specific assays. 2 6 In case this reference curve is unavailable, the following can be considered:

### TSH

By knowing that the TSH is low in the first trimester of pregnancy given the high concentrations of hCG and there are difficulties in obtaining a standardization, the lower limit of normality can be considered as 0.1 mIU/L. 7 9

From the second trimester onwards, TSH concentrations increase slightly, and the lower limit of normality in pregnant women can be considered the same used for non-pregnant individuals. 2 Patients diagnosed with GD hyperthyroidism have TSH < 0.1 mU/L and often undetectable TSH.

### T4T

The T4T concentration increases progressively until week 16, when it stabilizes influenced by the increase in TBG. From that period until delivery, consider 1.5 times the reference values provided by the laboratory for non-pregnant people. Therefore, a high concentration of T4T is expected during a normal pregnancy, that is, values at the upper limit or even above the usual reference values. 2 6 10

### FT4

The gold standard for dosing FT4 is by liquid chromatography mass spectrometry or equilibrium dialysis or ultrafiltration, which eliminate any interference. 2 However, these methodologies are costly and not available in most laboratories. The widely used automated immunoassays suffer interference from excess TBG, particularly from the second trimester, providing false low FT4 values.

Although there are difficulties both physiological of pregnancy and related to variations imposed by the laboratory method used, the best option for the diagnosis of overt hyperthyroidism in pregnant women is through elevated concentrations of FT4 above the upper limit of the laboratory reference considered, in conjunction with low or suppressed TSH.

## Should GTT be treated with ATDs?

Antithyroid drugs are not recommended for the treatment of GTT, because in this condition, spontaneous normalization of TH concentrations occurs until the end of the first trimester and may persist until week 18 of pregnancy, and these drugs are associated with congenital malformations and fetal hypothyroidism. 2

## How should the management in the face of a diagnosis of subclinical hyperthyroidism be?

Pregnant women with subclinical hyperthyroidism have TSH suppressed and thyroid hormone levels (FT4) within the reference value for pregnant women. The most important issue is to define the differential diagnosis between GTT and other forms of mild hyperthyroidism, among which the most common is GD. Whenever possible, this initial follow-up should be performed by the obstetrician in partnership with the endocrinologist. Careful anamnesis and physical examination with emphasis on GD stigmata (goiter and ophthalmopathy) should be performed, and the TSH and FT4 dosage should be repeated. The measurement of TRAb and T3 can help in etiological diagnosis. In GTT, the clinical picture is generally milder and may overlap with signs and symptoms of hyperemesis gravidarum (nausea and vomiting in early pregnancy with weight loss > 5%, dehydration and ketonuria); there is no previous history of thyroid disease and no GD stigmata such as goiter and ophthalmopathy. 9 However, GD can also manifest in a milder form during pregnancy, including subclinical hyperthyroidism; in this case, the patient usually has a previous history of thyroid disease, with or without a goiter and ophthalmopathy.

The presence of circulating TRAb confirms GD. 9

## What subsidiary exams should be requested to define the etiologic diagnosis of hyperthyroidism?

The etiological diagnosis of hyperthyroidism during pregnancy is essential, because it requires different treatments and has different prognoses ( Chart 2 ). The main diagnostic test is the TRAb measurement, particularly in the case of overt hyperthyroidism, since it is sensitive and specific for GD. In the absence of TRAb, an important increase also in T3 concentrations (total or free) in relation to T4 suggests GD. The level of T3 is slightly elevated in <20% in women affected by GTT. 10 11 Thyroid ultrasound may be useful when the etiologic suspicion is a toxic multinodular goiter or toxic adenoma (Plummer’s disease). 10 11 Scintigraphy is absolutely contraindicated in pregnancy. It is important to warn that the use of biotin should always be suspended before blood collection for these measurements, as most tests to assess thyroid function suffers interference, which mimics clinical hyperthyroidism with TSH suppression, FT4 elevation and positive TRAb. 12 It is also recommended to stop taking vitamins at least 48 hours before blood collection.

**Chart 2:** Differential diagnosis between GD and GTT

	Graves Disease	TTG
TRAb	Positive	Negative
T3	Elevated	Normal
T3:T4	Elevated	Low
T4L	Greatly increased	Slightly increased
TSH	Suppressed	Low or suppressed

GTT: gestational transient thyrotoxicosis; GD: Graves’ disease; TSH: thyroid stimulating hormone; TRAb: thyrotropin receptor antibody; T3: triiodothyronine; FT4: free thyroxine; T3:T4: ratio between T3 and T4 concentrations

Source: Prepared by the Working Group for Thyroid Dysfunctions in Pregnancy (CNEGAR and Brazilian Society of Endocrinology and Metabology – SBEM).

## Which patients with hyperthyroidism should be treated during pregnancy?

Overt hyperthyroidism is associated with unfavorable outcomes in pregnancy if left untreated. Poor thyrotoxicosis control is associated with fetal loss, gestational hypertension, prematurity, low birth weight, intrauterine growth restriction, thyrotoxic crisis, and maternal congestive heart failure. 13 Patients with overt hyperthyroidism should be treated, while those with subclinical hyperthyroidism and/or GTT should undergo symptomatic treatment and monitoring. 2

## How should care for patients diagnosed with subclinical hyperthyroidism be?

Since there is no evidence of worsening of maternal or fetal outcomes in pregnant women with subclinical hyperthyroidism, 11 14 ATD treatment is not recommended for them. Although worsening or progression to overt hyperthyroidism during pregnancy is uncommon, TSH and FT4 should be monitored every four weeks in order to identify patients who progress unfavorably. 2 The measurement of TRAb is important for the etiological diagnosis of subclinical hyperthyroidism. In case of positive TRAb, if concentrations are greater than three times the upper limit of normality, a new measurement should be taken between weeks 18-22, when the fetal thyroid is fully formed. Monitoring of TRAb should be performed as it is known to cross the placenta. 2 In cases of GD, special care should be taken after delivery given the higher recurrence of the disease in the postpartum period, and the patient should be referred to an endocrinology service for monitoring thyroid function during this period. Gestational transient thyrotoxicosis is another cause of subclinical hyperthyroidism in pregnancy, so thyroid function (TSH, FT4) should be monitored every four weeks until normalization occurs, usually around weeks 14-18. After normalization of thyroid function and if there are no associated diseases, the pregnant woman can undergo regular follow-up. Special attention should be taken when there is an association with hyperemesis gravidarum; in these cases, control of vomiting and intravenous hydration may be needed. Some cases may require hospitalization. 2 15 In cases of tachycardia with discomfort for the pregnant woman and elevated FT4, symptomatic drugs, such as beta-blockers, can be used. The medication of choice is propranolol, administered in doses of 10-40 mg every eight hours for a short time, until the FT4 level is normalized. 2 As prolonged use of propranolol during pregnancy has been associated with fetal bradycardia, neonatal hypoglycemia and fetal growth restriction, it should be used for the shortest possible time. 16

## What preconception care should be taken in women with hyperthyroidism?

Pregnancy planning should be discussed extensively between a woman of childbearing age with hyperthyroidism and her physician. It is important to inform the teratogenic and obstetric risks of hyperthyroidism, as well as its complex treatment during pregnancy. The institution of treatment is recommended, and the woman should be in stable euthyroidism on low doses of ATD (5-10 mg daily of MMZ or 50-200 mg of PTU) before considering conception. This state is considered when two consecutive tests with a minimum interval of one month in between show euthyroidism. 2 6 Particularly in conditions where the disease is difficult to control with ATDs, definitive treatment with ^131^ I or thyroidectomy should be offered prior to conception. It is important to remember that in cases of treatment with ^131^ I, even if control of hyperthyroidism is achieved in the short/medium term, there is a high chance of immunological worsening with high titers of circulating TRAb in response to therapy, which may last for a few months. Women who have been treated for at least six months with low doses of ATDs – 5-10 mg/day of MMZ or 100-200 mg/day of PTU – and are well controlled may have medication discontinuation considered in the first trimester, in view of the teratogenic potential of these drugs, provided they are regularly monitored for thyroid function. 7 17

## What should be the type of antenatal care follow up in pregnant women with hyperthyroidism?

Pregnant women with overt hyperthyroidism, in view of the complexity of medication adjustment and maternal and fetal risk, should be followed up in high-risk antenatal care. In this follow-up, permanent interaction between the obstetrician and endocrinologist is necessary, as well as special monitoring of the fetus. 2 6

## How should the drug treatment of hyperthyroidism diagnosed in pregnancy be?

**Figure 3.**
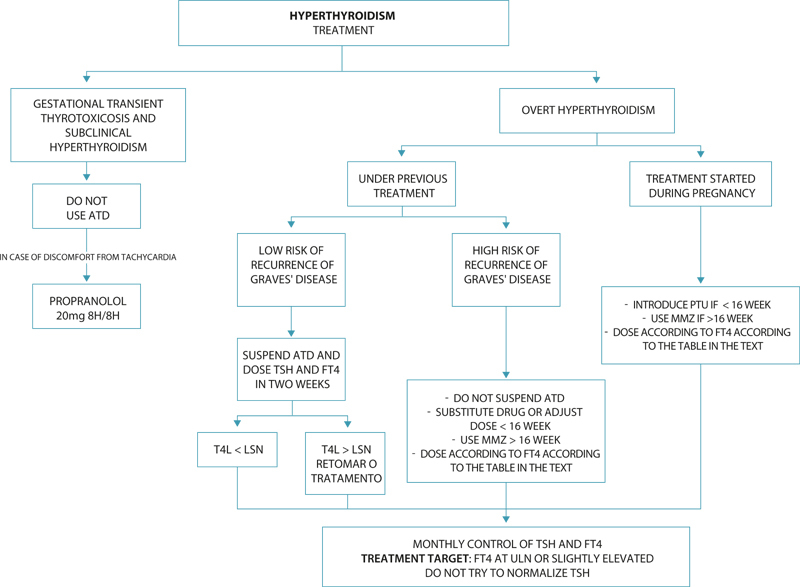
Hyperthyroidism - treatment. TSH: thyroid stimulating hormone; FT4: free thyroxine; ATD: antithyroid drug; PTU: propylthiouracil; MMZ: methimazole; ULN: upper limit of normality. Source: Prepared by the Working Group for Thyroid Dysfunctions in Pregnancy (CNEGAR and Brazilian Society of Endocrinology and Metabology – SBEM).

Propylthiouracil and MMZ are the drugs available for the treatment of hyperthyroidism during pregnancy. Because of adverse effects, especially the possibility of congenital malformations and fetal hypothyroidism, it is recommended to use the lowest possible dose, keeping the mother slightly thyrotoxic in order to preserve fetal thyroid function, as ATDs cross the placental barrier. 10 18 The dose depends on the level of FT4. Doses vary between 5-30 mg daily of MMZ (average of 10-20 mg), in a single daily dose, and between 100-600 mg of PTU daily (average of 200-400 mg), divided into two to three daily intakes. 2 Initial doses of ATDs should be proportional to the severity of thyrotoxicosis, as measured by the FT4 level. Chart 3 shows a suggestion of doses for MMZ and PTU adapted from the ATA guideline for the treatment of hyperthyroidism and thyrotoxicosis for non-pregnant women and adjusted to doses recommended for pregnant women ( Figure 3 ). 5 Propylthiouracil is the drug of choice when therapy is required up to week 16 of pregnancy, given the lower risk and lower severity of associated congenital malformations. In case it is necessary to maintain the ATD after week 16, PTU can be replaced by MMZ because of the higher risk of hepatotoxicity attributed to PTU. However, the change of medication can lead to uncontrolled hyperthyroidism. Thus, each pregnant woman should be evaluated individually to decide on the need to change medications. The dose equivalence between MMZ and PTU is 1:20 (5 mg of MMZ is equivalent to 100 mg of PTU). 2 If treatment begins after week 16 of pregnancy, it is recommended to start therapy with MMZ. 2 There is a tendency for improvement of GD during pregnancy due to immunological changes of this period and the increase in the TBG binding hormone; for this reason, ATD doses need to be reviewed at each visit, and a 30-50% dose reduction is recommended when FT4 level reaches the upper limit of normality. 5 The use of ^131^ I is contraindicated during pregnancy, as it crosses the placental barrier and causes fetal hypothyroidism and also by exposing the fetus to radiation. Thyroidectomy during pregnancy may be indicated if there are serious adverse effects related to ATDs and when the therapeutic goals of controlling hyperthyroidism cannot be achieved even with high doses of ATDs (>40 mg of MMZ or 600 mg of PTU). 2 If necessary, the best time for thyroidectomy is the second trimester of pregnancy. Preparation can be performed with beta-blockers. Special care should be taken in pregnant women with very high TRAb (>3 times the reference value), as the reduction in TRAb after surgery is slow and, even with successful control of maternal hyperthyroidism, there may still be a risk of fetal hyperthyroidism, requiring fetal monitoring. 2

**Chart 3:** Suggested MMZ and PTU doses for the start of treatment in pregnant women according to FT4 level

FT4 (number of times the upper normal limit)	METHIMAZOLE MG	PROPYLTHIOURACIL MG
Up to 2 times	5-10	100-200
2-3 times	10-20	200-400
Above 3 times	20-30	400-600

FT4: free thyroxine.

Source: Adapted from Ross et al. 5

## How should the treatment be for women who were already medicated for hyperthyroidism and get pregnant?

For patients at low risk for GD recurrence who are euthyroid and get pregnant while on low doses of ATDs (5-10 mg MMZ or 50-200 mg PTU), discontinuation of ATDs and observation of thyroid function are suggested. It is important to take into account other factors associated with disease recurrence, such as high levels of TRAb, use of ADT for less than six months, suppressed TSH while using ATD, voluminous goiter, presence of ophthalmopathy, and the need for higher doses than 5-10 mg MMZ or 50-200 mg PTU ( Chart 4 ). In cases of high risk for recurrence, the suggestion is to maintain PTU or change MMZ to PTU in the 1:20 ratio. Hyperthyroidism may improve during pregnancy, leading to the need for lower doses of ATDs or even discontinuation of medication. For this reason, dose adjustments based on serial laboratory evaluations should be made throughout pregnancy. 2

**Chart 4:** High-risk criteria for GD recurrence after withdrawal of ATDs in pregnant women

Elevated TRAb levels (3 times the upper limit of normal)
Use of ATDs for less than 6 months
TSH suppressed in use of ATDs
Voluminous goiter
Graves’ ophthalmopathy
Need for higher doses than 5-10 mg of MMZ or 50-200 mg of PTU

MMZ: methimazole; PTU: propylthiouracil; GD: Graves’ disease; ATDs: antithyroid drugs; TRAb: anti-TSH receptor antibody; TSH: thyroid stimulating hormone.

Source: Prepared by the Working Group for Thyroid Dysfunctions in Pregnancy (CNEGAR and Brazilian Society of Endocrinology and Metabology – SBEM).

## What are the maternal-fetal adverse effects of antithyroid drug treatment?

Antithyroid drugs are the mainstay of hyperthyroidism treatment in pregnancy. The most used drugs are PTU and MMZ, and both fundamentally interfere in the process of thyroid hormone formation, inhibiting its synthesis. However, they can produce undesirable effects. Side effects occur in 3-5% of mothers and are mostly eruptive allergic reactions or gastrointestinal symptoms, which can be controlled with symptomatic drugs (antihistamines) or by switching between ATDs. However, in 0.1-0.15%, they can lead to serious effects such as agranulocytosis and liver failure. These two effects are an absolute contraindication for the use of ATDs and if they occur with the use of one drug, switching to the other is not recommended. The patient should be urgently referred to a specialized service, and thyroidectomy can be considered, ideally in the second trimester. All pregnant women using ATDs should be warned that in the presence of flu-like signs and/or symptoms such as fever and odynophagia, there is a possibility of agranulocytosis, and blood count should be collected, ATD discontinued and antibiotic therapy started immediately if the diagnosis is confirmed. 17 19 The effects on the fetus are due to the transplacental passage of these medications. In the first trimester, we are faced with teratogenic effects. Methimazole leads to complications in 2-4% of pregnancies. The best known malformation is aplasia cutis, although other malformations have been described, namely cloacal and esophageal atresia, defects in the formation of the abdominal wall and malformations of the eyes, heart and urinary system. Propylthiouracil can also lead to fetal malformations in 2-3%. The malformations we can find are cervical cysts and male urinary tract abnormalities. Note that malformations related to PTU are usually less severe than those triggered by MMZ. 19 20 21 In addition to interfering with fetal formation, when these medications cross the placental barrier, they can interfere with fetal thyroid hormone synthesis, causing fetal hypothyroidism. Thus, precise management of these medications during pregnancy is essential. The dose of ATDs should be the minimum necessary. Propranolol can also be used to control the symptoms of hyperthyroidism. If this use is extended over long periods, it can cause fetal growth restriction, fetal bradycardia and neonatal hypoglycemia. 21

## How to monitor the treatment for hyperthyroidism and what is the laboratory goal to be achieved?

The goal during treatment of hyperthyroidism in pregnancy is to maintain FT4 concentrations at the upper limit of normal or slightly elevated. This way, excessive treatment with ATDs will be avoided and consequently, fetal hypothyroidism. Monitoring should be done every 2-4 weeks until the pregnant woman is on a stable dose of ATD. Then, follow-up can be done every four weeks. The TSH can remain low or undetectable for weeks, even after FT4 has decreased. Therefore, TSH should not be a follow-up parameter during treatment. Hyperthyroidism in pregnant women will be well controlled in the presence of FT4 at the upper limit of normal (or slightly elevated), even with TSH still low. 2 8 Patients with GD should measure TRAb in the first trimester. If concentrations are greater than three times the upper limit of normal, a new measurement should be taken between weeks 18-22, when the fetal thyroid is fully formed. The TRAb level should be monitored as it is known to cross the placenta. If high TRAb titers persist, fetal goiter assessment and monitoring should be performed given the higher risk of fetal and neonatal hyperthyroidism. If titers become negative or decrease, the risk of maternal-fetal complications is lower. 2 22

## How and at what intervals should maternal-fetal follow-up be performed?

**Figure 4.**
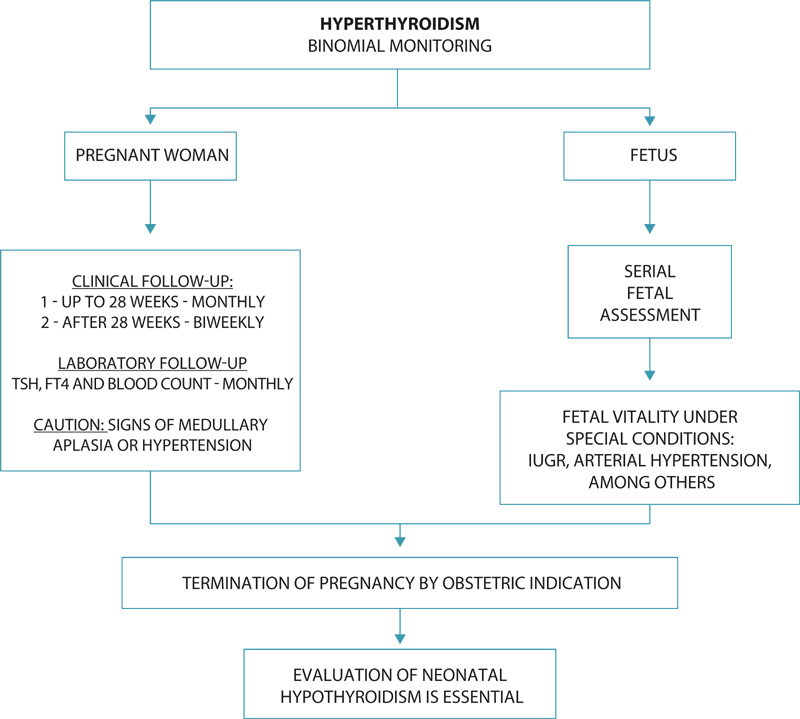
Hyperthyroidism – Monitoring the binomial. TSH: thyroid stimulating hormone; FT4: free thyroxine; IUGR: intrauterine growth restriction. Source: Prepared by the Working Group for Thyroid Dysfunctions in Pregnancy (CNEGAR and Brazilian Society of Endocrinology and Metabology – SBEM).

As a general rule, multidisciplinary follow-up involving the obstetrician and endocrinologist in high-risk antenatal care is advised. Appointments should be carried out every other week until week 28, and weekly until delivery. The fetus should be monitored for formation, growth, and signs of hypothyroidism or hyperthyroidism throughout pregnancy. After week 28, in specific situations – such as fetal growth restriction – it is recommended to include periodic assessments of fetal vitality ( Figure 4 ). 5 6

## How to diagnose and manage fetal hyperthyroidism or hypothyroidism?

Fetal hyperthyroidism typically manifests with persistent fetal tachycardia (FHR > 170 bpm) for more than 10 minutes in pregnant women with GD. Other fetal clinical signs can be: fetal growth restriction, fetal goiter, accelerated bone maturation and in more extreme cases, congestive heart failure and hydrops. Fetal hyperthyroidism is triggered by the passage of TRAb through the placental barrier, excessively stimulating the formation and release of THs from the fetus. These pregnant women must be monitored in a particular way by an experienced fetal medicine team. This condition can even extend to the neonatal period, and there must be strict monitoring by the obstetrician and neonatologist. A condition that should be considered as high risk is the scenario of women with a previous diagnosis of GD who underwent ^131^ I or thyroidectomy prior to pregnancy. As these women no longer use ATDs, they may continue to produce TRAb, causing fetal and/or neonatal hyperthyroidism. 6 23 24 Fetal hypothyroidism is usually manifested by fetal goiter, as a result of the passage of ATDs or rarely, by the passage of inhibitory TRAb, which would decrease the production of THs by the fetus. The pregnant woman must be accompanied by an experienced fetal medicine team. Cordocentesis or the measurement of THs in amniotic fluid for the diagnosis of fetal hypothyroidism is extremely controversial, as is the administration of levothyroxine in amniotic fluid. 6 In case of massive fetal goiter, the need for EXIT (ex-utero intrapartum treatment) at delivery should be discussed.

## How to prescribe treatment for hyperthyroidism during lactation?

Antithyroid drugs are secreted into breast milk, although in very low concentrations. Breastfeeding is safe with doses up to 20 mg/day of MMZ and 450 mg/day of PTU. 2 Antithyroid drugs should be taken immediately after breastfeeding. 10 The preference for MMZ is due to the side effects associated with PTU. 5 Monitoring the infant’s thyroid function is not necessary, as there is no evidence that ATD use leads to hypothyroidism or impairment in the child’s growth and neurocognitive development. 25 Since there are not enough data showing that hyperthyroidism interferes with lactation, it should be treated according to its diagnosis. 2 Treatment with ^131^ I can only be indicated after breastfeeding has been discontinued for at least three months and breastfeeding should not be resumed, 26 as the concentration of radioactive iodine in the mammary gland may increase the risk for future breast cancer. 27 Propranolol can be used during breastfeeding without risk to the child or interference with lactation ability. However, atenolol is not recommended as it is secreted into breast milk, causing bradycardia and hypoglycemia in the infant. 28

## How should care to postpartum women with thyrotoxicosis be?

Thyrotoxicosis that appears in the postpartum period is often caused by postpartum thyroiditis (PPT), defined as autoimmune thyroid dysfunction in the first year postpartum, excluding GD. 8 25 Another possibility is the recurrence or activation of GD in the postpartum period. It is important to differentiate between these two main causes. The incidence of PPT is extremely variable, reaching about 15% of cases. 2 It most often affects women with a previous history of PPT, who have positive antithyroid antibodies before pregnancy (under treatment with levothyroxine or in euthyroidism), with another associated autoimmune disease (insulin-dependent diabetes, rheumatoid arthritis, etc.) or even with GD in remission. 29 The typical clinical picture begins with a toxic phase, clinical and laboratory hyperthyroidism, followed by hypothyroidism and later recovery of thyroid function. Thyroid function shows suppressed TSH and elevated FT4 level, defining thyrotoxicosis (toxic phase) and the presence of antithyroid antibodies, particularly the antithyroperoxidase (anti-TPO) antibody, characterizes PPT. The thyrotoxicosis phase is usually mild and self-limiting, lasting from one to three months, when the patient may experience palpitations, tremors, fatigue and nervousness. At this stage, it is important to differentiate it from GD triggered in the postpartum period ( Figure 1 ) ( Chart 5 ). The hyperthyroid phase of PPT usually appears in the first few months after delivery, while GD can appear after this period (three months after delivery). 30 Given the destructive process of PPT, higher concentrations of T4 are observed in relation to T3, while in GD, there is a predominance of higher concentrations of T3 than T4. The presence of TRAb favors the diagnosis of GD. Scintigraphy should be avoided due to the passage of the radioisotope into breast milk. If it is essential for the diagnosis, the option is to use 99mTc or ^123^ I, with shorter half-lives, and breast milk should be removed and discarded for 1-5 days, respectively, until breastfeeding is resumed. 31 Treatment of the toxic phase of PPT can be instituted to mitigate the clinical picture by using beta-blockers, which are not contraindicated during breastfeeding. The dose of propranolol is given according to symptoms, starting with 10mg three times a day. Atenolol, on the other hand, should be avoided, as it is secreted into breast milk, causing bradycardia and hypoglycemia in the infant. The use of ATDs is contraindicated in PPT. 2 Monitoring with TSH and FT4 should be performed every 4-8 weeks. 29 The concern after the hyperthyroid phase is hypothyroidism, which may be more symptomatic, occurring 3-12 months after delivery. 2 Treatment with levothyroxine should be started if the patient has significant symptoms, if she is breastfeeding, if the TSH elevation continues for more than six months, and especially if she is planning a new pregnancy. Levothyroxine withdrawal can be planned after 6-12 months of treatment. Monitoring of TSH and FT4 should be done annually given the high risk of developing permanent hypothyroidism after PPT. 2 6

**Chart 5:** Differential diagnosis between postpartum thyroiditis and Graves’ disease

	Postpartum thyroiditis	Graves disease
Onset after delivery	<3 to 6 months	>3 to 6 months
TRAb	Negative	Positive
Anti-TPO	Present	May be present
T3:T4	Low (T4 >>T3)	Elevated (T3>>T4)
Thyroid vascularization	Normal	Increased

TRAb: anti-TSH receptor antibody; anti-TPO: antithyroperoxidase antibody; T3:T4: ratio of triiodothyronine and thyroxine concentrations.

Source: Prepared by the Working Group for Thyroid Dysfunctions in Pregnancy (CNEGAR and Brazilian Society of Endocrinology and Metabology – SBEM).

## Final considerations

Hyperthyroidism in pregnancy represents a major challenge for both the obstetrician and the endocrinologist. Pregnancy changes in thyroid hormone physiology, fetal, neonatal and maternal complications of untreated disease, fetal and maternal repercussions, as well as the details of drug treatment during pregnancy are important aspects that should be on the radar of this follow-up treatment. In the first trimester of pregnancy, gestational thyrotoxicosis, present in up to 5% of pregnancies, stands out. Its etiopathogenesis is primarily related to the production of hCG, leads to symptoms usually more related to hyperemesis gravidarum and does not require treatment with antithyroid drugs. An important aspect is the differentiation with GD diagnosed during pregnancy. In general, GD is the most associated pathology with hyperthyroidism in pregnancy. This aspect also brings the effects of TRAb on fetal thyroid function, as these antibodies cross the placental barrier. Untreated hyperthyroidism is linked to a series of complications, such as: fetal loss, hypertension, prematurity, fetal growth restriction, fetal hyperthyroidism, thyrotoxic crisis, maternal congestive heart failure and maternal death, the latter being rare. Thus, adequate treatment of hyperthyroidism during pregnancy is important. However, the drugs from the thiamine class available for this treatment, PTU and MMZ, are potential teratogenic substances that can cause fetal hypothyroidism and goiter, and are related to several side effects in pregnant women, some very serious, such as hepatitis and pancytopenia. Other more effective treatments, such as ^131^ I, are proscribed during pregnancy and breastfeeding as they can lead to fetal and neonatal thyroid injury. Surgery represents a greater risk in pregnancy and should be indicated in very specific situations. With all this dynamics, drug treatment with the lowest possible dose of medication is necessary during pregnancy. Systematic laboratory control of collateral and fetal effects and careful obstetric follow-up are also essential. Childbirth does not necessarily represent a relief, as the rebound of the autoimmune system - suppressed during pregnancy - and the return to normal conditions of production and peripheral transport of TH can trigger new cases of GD and worsen the existing GD. Finally, it is important to emphasize that drug treatment of hyperthyroidism does not contraindicate breastfeeding, but additional care must be taken.

**National Commission Specialized in High Risk Pregnancy of the Brazilian Federation of Gynecology and Obstetrics Associations (Febrasgo)**

President:

Rosiane Mattar

Vice-president:

Alberto Carlos Moreno Zaconeta

Secretary:

Mylene Martins Lavado

Members:

Maria Rita de Figueiredo Lemos Bortolotto

Fernanda Santos Grossi

Vera Therezinha Medeiros Borges

Inessa Beraldo de Andrade Bonomi

Janete Vettorazzi

Carlos Alberto Maganha

Renato Teixeira Souza

Felipe Favorette Campanharo

Sara Toassa Gomes Solha

Arlley Cleverson Belo da Silva

Elton Carlos Ferreira

**Thyroid Department of the Brazilian Society of Endocrinology and Metabology (SBEM)**

President:

Patrícia de Fátima dos Santos Teixeira (RJ)

Vice-president:

Danilo Glauco Pereira Villagelin Neto (SP)

Directors:

Rafael Selbach Scheffel (RS)

Cléo Otaviano Mesa Júnior (PR)

Gláucia Maria Ferreira da Silva Mazeto (SP)

Maria Izabel Chiamolera (SP)

Helton Estrela Ramos (BA)
